# Self-Assembled Monolayers of Various Alkyl-Phosphonic Acids on Bioactive FHA Coating for Improving Surface Stability and Corrosion Resistance of Biodegradable AZ91D Mg Alloy

**DOI:** 10.3390/ma18194633

**Published:** 2025-10-08

**Authors:** Chung-Wei Yang, Peng-Hsiu Li

**Affiliations:** Department of Materials Science and Engineering, National Formosa University, No. 64, Wunhua Road, Huwei, Yunlin 632301, Taiwan

**Keywords:** Mg alloys, FHA coatings, self-assembly monolayers, surface chemistry, XPS analysis, corrosion resistance

## Abstract

The aim of present study is to deposit protective coatings with various surface chemical states on AZ91D Mg alloy. Hydrothermal bioactive ceramic coatings are performed with a surface modification by the chemical bonding of self-assembled monolayers (SAM). The electrochemical corrosion behaviors of various surface-coated AZ91D alloy within DMEM cell culture medium related to their surface chemical states are evaluated through microstructure observations, XPS surface chemical bonding analysis, static contact angles measurements, potentiodynamic polarization curves, and immersion tests. XRD and high resolution XPS of F 1*s* analysis results show that the hydrothermal FHA coating with a phase composition of Ca_10_(PO_4_)_6_(OH)F can be effectively and uniformly deposited on the AZ91D alloy. FHA-coated AZ91D displays better anti-corrosion performances and lower degradation rates than those of uncoated AZ91D alloy in the DMEM solution. Through the high resolution XPS analysis of O 1*s* and P 2*p* spectra, it is demonstrated that 1-butylphosphonic acid (BP), 1 octylphosphonic acid (OP), and dodecylphosphonic acid (DP) molecules can be effectively bonded on the FHA surface by a covalent bond to form SAM. BP/OP/DP-SAM specimens display increased static contact angles to show a hydrophobic surface. It demonstrates that the SAM surface treatment can further enhance the corrosion resistance of FHA-coated AZ91D in the DMEM solution. After 2–16 days in vitro immersion tests in the DMEM, the surface SAM-bonded hydrophobic BP/OP/DP-SAM layers can effectively inhibit and reduce the penetration of DMEM into FHA coating. Long alkyl chains of the dodecylphosphonic acid (DP) SAM represents superior enhancing effects on the reduction of corrosion properties and weight loss.

## 1. Introduction

Magnesium-based (Mg) alloys have become a new generation of biomedical metallic materials due to their high chemical activity and biodegradable properties [1,2]. They have been widely studied and applied as biodegradable scaffolds in the field of biomedical tissue engineering [3,4,5]. In medical engineering and clinical applications, Mg alloys are also used as cardiovascular devices, hard tissue replacement bone nails and bone plates, regeneration therapies, and bone graft scaffolds [2,6,7,8]. In addition to being able to be eventually absorbed by the human body through degradation, Mg alloys also have biological activity to stimulate new bone cell proliferation and attachment, thereby inducing the repair of bone tissues. The Mg-Al-Zn alloy system, which is known as the AZ-series alloy, is the most commonly used Mg alloys. The addition of Al and Zn elements can help to increase the castability, corrosion resistance and mechanical strength through the combination of solid solution strengthening, and precipitation hardening effects. Zn also assists in overcoming harmful corrosive effects of impurity in Fe and Ni elements. The die-cast AZ91D alloy (generally contains Al about 8.3–9.7 wt.% and Zn about 0.45–0.9 wt.%) is the most frequently used commercial Mg-Al-Zn alloy due to its high strength and relatively good corrosion resistance [9], and the AZ91D alloy is selected as the substrate in this study. However, several studies indicated that Mg alloys still undergo varying degrees of corrosion from moderate to severe, depending on alloy chemistry, under physiological conditions [8,10,11,12,13]. Therefore, the degradation rate of Mg alloys must be reduced to retain mechanical and structural integrity of implants to keep intact before sufficient healing of new tissues. Surface treatments to improve the corrosion resistance of biomedical Mg alloys in physiological environment solutions have been a research focus in the applications of Mg alloys as biodegradable implant materials.

The release of Mg^2+^ ions during the degradation of Mg alloys generally influences biocompatibility, cells adhesion, cells viability, cytotoxicity, and interfacial affinity between tissues and implants [13,14]. Therefore, it is important to understand surface chemical reactivity for the Mg alloy. Factors affecting the biocompatibility of medical hard tissue replacement materials include surface topography, surface roughness, surface free energy, and surface chemistry [15]. Researchers tried to develop the highly biocompatible surface to reduce the degradation rate [16,17] and enhance the osteogenesis reactions [18,19] for the biomedical stability of Mg-based implants. Deposition of bioactive calcium phosphate coatings is an effective method to improve the corrosion resistance of Mg alloys [20,21,22,23,24,25,26] because they can provide a barrier between the Mg substrates and the corrosion medium. Hydroxyapatite (HA, Ca_10_(PO_4_)_6_(OH)_2_), whose chemical component is quite similar to major inorganic constituents of human hard tissues, is a common bioactive ceramic of the calcium phosphates due to its good biocompatibility and osteoconductive properties. It has also been widely investigated as protective coatings to improve biological properties and corrosion resistance of metallic implants in dentistry and orthopedics. Surface HA coatings, which are deposited on Mg substrates by a chemical solution deposition method, were shown to inhibit corrosion [27]. Fluorine (F) is an essential trace element against dissolution of human hard tissues. In order to enhance the chemical stability and dissolution resistance of HA, doping of fluoride (F^−^) ions in the HA lattice is developed and studied [28,29,30], and fluoridated HA (FHA, Ca_10_(PO_4_)_6_(OH)_2−x_F_x_) has been developed as a more promising bioactive ceramic than the stoichiometric HA for dentistry and orthopedic applications. Shen et al. indicated that FHA coating with nano-topography prepared on AZ31 Mg alloy significantly enhanced osteogenic differentiation capacity compared to the HA coating [28]. Razavi et al. prepared nanostructured fluoridated hydroxyapatite bioceramic coating by electrophoretic deposition (EPD), which also improves the corrosion resistance and biological properties of Mg alloys [31]. Gao et al. studied the surface of Mg alloy coated with calcium phosphate coating and found that bone cells had good adhesion and fast cell growth and proliferation rates after bone cell culture experiments [27]. Animal experiments also confirmed that this surface layer has significant ability to guide bone formation, and there is obvious osteogenesis after 4 weeks of implantation. Our previous study demonstrated that fluoridated hydroxyapatite-coated Mg scaffolds can significantly reduce the degradation rate of AZ80 Mg alloy, and exhibits a time-dependent, stable release of Mg^2+^ ions [32]. However, even the same type of surface morphologies has different chemical states on the basis of their fabrication method to influence their bone cell response [33].

Chemical modification of a metallic substrate using organic compounds is a current active research subject in biotechnology to control surface chemical states and improve corrosion resistance for biodegradable Mg alloys. The concept of decreasing corrosion rate of Mg alloys is to deposit a hydrophobic film by chemical treatments because the hydrophobic surface can reduce the direct contact of environmental humidity and corrosive containing solutions. The deposition technique of self-assembled monolayers (SAM) can prepare a well-defined surface chemistry for the substrates and has been adopted to study the effect of organic functional groups graft on calcium phosphate coatings and metallic substrates [34,35,36,37,38]. Self-assembled monolayers are thin organic modification layers formed on the top of a substrate. The SAM are typically long chain surfactant molecules, which have a chemical functionality at one end called the head-group to provide a specific chemical affinity towards substrates, and are attached by chemisorption. The other end of the long chain molecule also has a function and is called the terminal group (or the end group). The terminal group determines the surface characteristics of the SAM system and any further modification of the surface. Methyl (–CH_3_), carboxyl (–COOH), and amine-terminated (–NH_2_) are generally used terminal functional groups of the SAM molecules [34,38]. The terminal amine group provides a positively charged surface, the terminal carboxyl group provides a negatively charged surface, and the terminal methyl group provides a neutral surface of substrates. Since the molecular structure of the alkyl-phosphonic acids displays an alkyl chain, the purpose of using organic alkyl-phosphonic acids (RPO(OH)_2_, R: alkyl group), therefore, is to provide a neutral surface of the SAM-treated specimens in this study. The Langmuir–Blodgett method [39] and the tethering by aggregation and growth (T-BAG) method [40] are typical surface grafting methods to modify the surface chemical status of metallic substrates. The advantage of T-BAG method to prepare SAM is that the organic functional groups can directly chemically bond to the substrates without promoting surface activation or applying pressure. The concept for preparing SAM is based on the surface charge of an inorganic material (metal/ceramic), which spontaneously forms chemical bonds with the active functional groups of the organic compound through hydrogen bonds, van der Waals forces, or other non-covalent bonds between the surfactant molecules and substrates [38]. In this method, the substrate is immersed in a solution containing organic surfactant molecules. After immersion for a period of time, the organic surfactant molecules are then adsorbed and self-assembled on the surface to form regularly two-dimensional arranged and tightly ordered monolayers. These surfactant organic molecules typically have long carbon chains, with head groups covalently bonded to the material surface and leaving terminal groups exposed. The surface chemical properties are then modified by the specific structural characteristics of the terminal functional groups. Some organic alkanoic and phosphonic acids grafted SAMs are applied as the chemical modification surface layers on Mg alloys by liquid/vapor phase methods to improve their chemical stability [41,42]. Whitesides et al. [43] and Arima et al. [44] indicated that the grafting of SAM on the surface of biomaterials obviously enhances the adsorption of fibronectin, laminin, albumin, and collagen in the extracellular matrix. The SAM is thought of as a potential technique for controlling surface chemistry to improve the electrochemical corrosion resistance of Mg alloys and provide specific surface activity of engineering materials [37,41].

In the present study, a bioactive fluorohydroxyapatite (FHA) ceramic coating was first deposited on the AZ91D Mg alloy by hydrothermal synthesis. Following the hydrothermal deposition of FHA coatings, SAM surface treatments were performed by the T-BAG method with different alkyl chains length of the organic alkyl-phosphonic acids to change the chemical states for the surface of FHA-coated AZ91D specimens. The aim of present study is to improve the chemical stability and enhance the corrosion resistance of the biodegradable AZ91D alloy in a physiological medium. The phase composition and surface chemical states of depositing FHA-coated AZ91D with SAM were analyzed using X-ray diffraction (XRD) and X-ray photoelectron spectroscopy (XPS), respectively. The degradation behaviors and electrochemical properties were tested through immersion and potentiodynamic polarization experiments in a Dulbecco’s modified eagle medium (DMEM).

## 2. Materials and Methods

### 2.1. Preparation of Hydrothermal FHA Coatings and the SAM Treatment

Commercial die-cast AZ91D Mg-based alloy (chemical composition in wt.%: 8.93% Al, 0.65% Zn, 0.18% Mn, 0.0168% Si, 0.05% Fe, 0.03% Cu, and the balance is Mg) with dimensions of 30 (*l*) × 10 (*w*) × 3 (*t*) mm was prepared as the substrate for the hydrothermal surface coatings synthesis. Prior to the preparation of hydrothermal surface coatings, AZ91D substrates were ground using 2000-grit SiC abrasive papers and then grit-blasted by Al_2_O_3_ particles (355–425 μm) to roughen the surfaces. The average surface roughness (Ra) of grit-blasted AZ91D substrates was measured about 4.8 ± 0.6 μm. The substrates were then ultrasonically cleaned in absolute ethanol for 10 min to remove surface impurity contaminations and air-dried for the following hydrothermal coating process. Analytical grade of dicalcium phosphate dihydrate (DCPD, CaHPO_4_·2H_2_O, 172.09, AR, 98 wt.%, SHOWA), calcium hydroxide (Ca(OH)_2_, 74.09, AR, 96 wt.%, Berlin), and hexafluorophosphoric acid (HPF_6_, 145.97, AR, 96 wt.%, ACROS) were used as raw materials for the hydrothermal synthesis of fluorohydroxyapatite (FHA, Ca_10_(PO_4_)_6_(OH)F) coatings. First, a solution containing DCPD and Ca(OH)_2_ powder mixtures was prepared with a Ca/P molar ratio of about 1.67. Subsequently, HPF_6_ solution with a concentration of 3 M was added into prepared aqueous solution of DCPD/Ca(OH)_2_ mixtures with stirring for 15 min. The final mixed solution with a pH value of 12 was prepared for the hydrothermal synthesizing FHA coatings. Then the hydrothermal treatment for synthesizing FHA coatings on AZ91D substrates was performed at 175 °C and held for 2 h in an autoclave.

Analytical grade of 1-butylphosphonic acid (BP, CH_3_(CH_2_)_3_PO(OH)_2_, 98%, Alfa Aesar, Thermo Fisher Scientific, Ward Hill, MA, USA), 1-octylphosphonic acid (OP, CH_3_(CH_2_)_7_PO(OH)_2_, 98%, Alfa Aesar), and dodecylphosphonic acid (DP, CH_3_(CH_2_)_11_PO(OH)_2_, 95%, Alfa Aesar) were applied for preparing self-assembled monolayers (SAM) bonded directly to the surface of hydrothermal FHA coatings using the T-BAG method on the basis of our previous study [45]. The FHA-coated specimens were immersed in a solution of the BP, OP, and DP acids (each concentration was 6 mM in absolute ethanol), respectively. The solvent was allowed to evaporate slowly for 7 h, until the level of solutions fell below the FHA-coated specimens. As the meniscus traverses to the FHA-coated specimens, BP, OP, and DP acids were transferred to the surface of FHA coatings. Then the SAM-treated FHA specimens were ultrasonically cleaned in absolute ethanol, and were heated at 120 °C in a vacuum oven for 12 h to bond BP, OP, and DP acids SAM to the surface of FHA specimens. In the following sections, these SAM surface-treated FHA specimens by the BP, OP, and DP acids will be denoted as “FHA-BP”, “FHA-OP”, and “FHA-DP”, respectively.

### 2.2. Microstructure Characterization and Analysis

The phase compositions of AZ91D substrate and hydrothermal FHA-coated samples were identified by an X-ray diffractometer (XRD, Bruker, D8A25, Bruker Corp., Karlsruhe, Germany), using Cu Kα radiation, operated at 40 kV and 40 mA over a range of 20–60° (2θ) with a scan speed of 3° min^−1^. Surface microstructural features of hydrothermal FHA coatings, FHA-BP, FHA-OP, and FHA-DP specimens were analyzed by a scanning electron microscopy (SEM, JEOL/JSM-6360, JEOL Ltd., Tokyo, Japan). Surface roughness of AZ91D substrates and various coatings were evaluated using a surface profilometer (Polytec, MSA-500 Stylus Prof, Kosaka Laboratory, Tokyo, Japan). The assessment of static contact angle (δ) was performed for the wettability of the specimens with various surface chemical states. All the specimens were cleaned with absolute ethanol, dried in N_2_ gas, and then the static contact angle was measured using 20 μL Dulbecco’s modified eagle medium (DMEM, Gibco, Thermo Fisher Scientific, Ward Hill, MA, USA) solution as the solvent. The measurements of static contact angles were conducted at room temperature.

Surface chemical states of AZ91D substrates, FHA coatings, FHA-BP, FHA-OP, and FHA-DP specimens were determined by X-ray photoelectron spectroscopy (XPS, PHI 5000 Versa Probe, ULVAC-PHI, Inc., Chigasaki, Kanagawa, Japan) using a monochromatic Al Kα radiation (*hν* = 1486.7 eV) at a base pressure of 1 × 10^−10^ torr. The measured binding energy (BE) was calibrated with reference to the adventitious C 1*s* at 284.8 eV. Survey spectra and high-resolution spectra of F 1*s*, O 1*s* and P 2*p* regions were obtained for each specimen. The Gaussian peak-fitting routine was performed in the analysis of high-resolution spectra for separating species in various chemical bondings.

### 2.3. Electrochemical Corrosion Behavior and the Immersion Tests

The electrochemical properties of AZ91D Mg alloys, hydrothermal FHA coatings, FHA-BP, FHA-OP, and FHA-DP specimens were investigated by potentiodynamic polarization tests using a potentiostat (SP-150, Bio-Logic Science Instruments, rue de Vaucanson, France) according to the ASTM G102-89. The corrosion cell applied in the electrochemical experiments was a conventional three-electrode electrochemical assembly, in which the Pt sheet and the saturated calomel electrode (SCE, Hg/Hg_2_Cl_2_) were used as the counter electrode and the reference electrode, respectively. Rectangular AZ91D and different surface-treated specimens with an exposed surface area of 100 mm^2^ were applied as the working electrode. All experiments were performed in the DMEM solution at 37 °C. Table 1 lists a comparison of ion concentrations and pH values for the DMEM solution and human blood plasma. Prior to starting of the experiments, the open-circuit potential (OCP) was measured in the corrosion cell containing DMEM solution. The specimens were then stabilized the open-circuit potential for 30 min in the DMEM solution, and the potentiodynamic polarization tests were performed at a constant scan rate of 0.5 mV/s. Corrosion potentials (*E*_corr._, V_SCE_) and corrosion current densities (*I*_corr._, μA/cm^2^) were determined from potentiodynamic polarization curves by the Tafel slope extrapolation method. The corrosion rate (CR, *P_i_*, mm/year) evaluated by the corrosion current densities was determined from the relation of *P_i_* = 22.85 × *I*_corr_.

Long-term degradation behaviors of the hydrothermal FHA coatings, FHA-BP, FHA-OP, and FHA-DP specimens within the DMEM solution were examined by the static immersion tests at 37 °C for different durations of 2, 4, 8, and 16 days according to the ASTM G31-72. First, the samples were weighed (*W_b_*, mg) before immersion. After immersion tests, samples were taken out of the DMEM solution at each defined time point. Each sample was cleaned with distilled water, dried for 24 h, and weighed (*W_a_*, mg) to obtain the immersed weight. Then the weight loss (*W_L_*, mg cm^−2^ d^−1^) of immersed samples was evaluated based on the following equation, where *A* and *t* denoted the surface area (mm^2^) of the sample and the immersion duration (d), respectively. The measurement of weight loss for every immersion duration was an average of five samples (*n* = 5).

(1) $$W_{L}=\frac{W_{b}-W_{a}}{A\cdot t}$$

## 3. Results and Discussion

### 3.1. Microstructural Features and Phase Compositions

**Figure 1:**
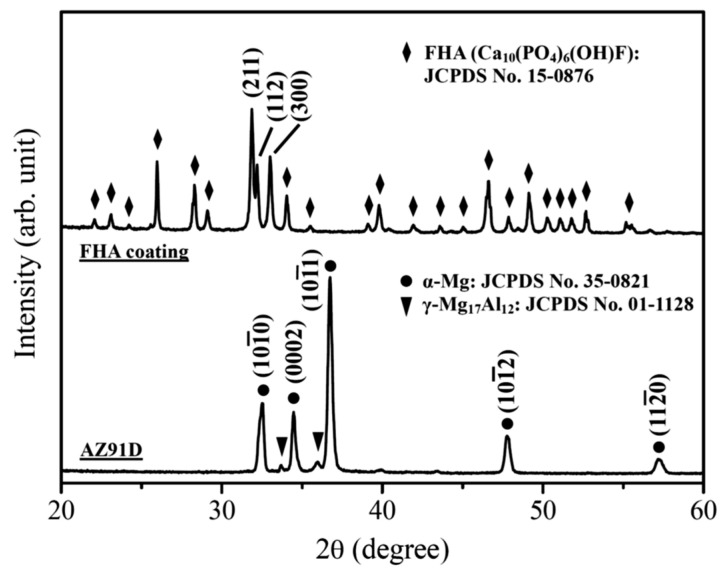
X-ray diffraction patterns of the AZ91D Mg substrate and hydrothermally synthesized FHA surface coatings.

Figure 1 shows the XRD patterns of the AZ91D Mg alloy and hydrothermally synthesized FHA coatings. The XRD pattern of AZ91D displays typical diffraction peaks of α-Mg phase (main diffraction peaks at 2θ = 32.6°, 34.9°, 37.2°, 48.5°, and 58.0°, JCPDS No. 35-0821). In addition, the identified γ-Mg_17_Al_12_ (diffraction peaks detected at 2θ = 33.9° and 36.1°, JCPDS No. 73-1148) is a common intermetallic compound (IMC) for AZ91D Mg alloy. Hydrothermally synthesized FHA coating is predominantly composed of a well-crystallized fluorohydroxyapatite phase (Ca_10_(PO_4_)_6_(OH)F, main diffraction peaks of (211), (112), and (300) lattice planes detected at 2θ = 31.9°, 32.3°, and 33.1°, respectively, JCPDS No. 15-0876) without other calcium phosphate impurity phases.

**Figure 2:**
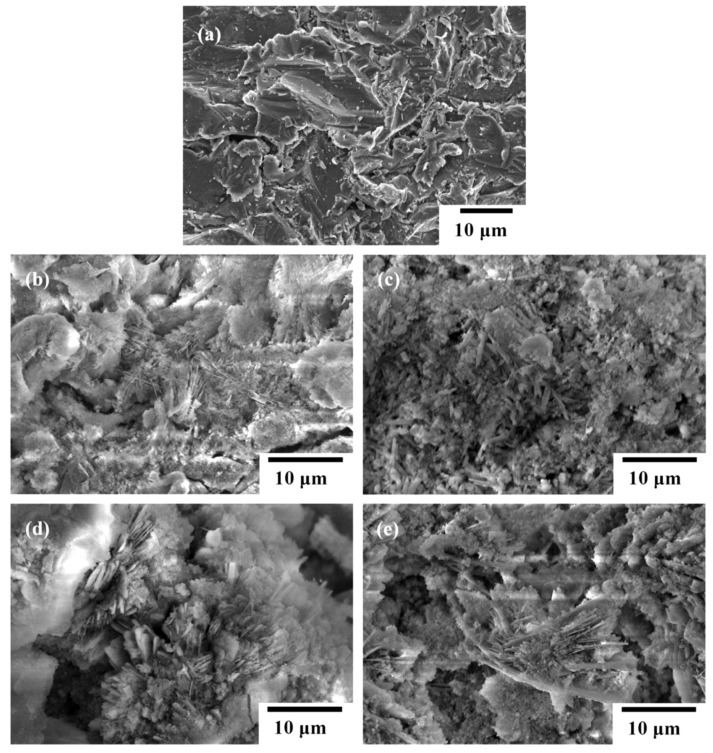
SEM surface micrographs of the (**a**) grit-blasted AZ91D Mg alloy, (**b**) hydrothermal FHA coatings, (**c**) FHA-BP, (**d**) FHA-OP, and (**e**) FHA-DP specimens.

Figure 2a shows the representative SEM surface micrographs of the grit-blasted AZ91D Mg substrate. Surface roughness (Ra, μm) of the grit-blasted substrate is about 4.8 ± 0.6 μm. Figure 2b–e show the representative SEM surface morphologies of the hydrothermal FHA coatings, FHA-BP, FHA-OP, and FHA-DP specimens, respectively. Table 2 also lists the average surface roughness (Ra, μm) of FHA-coated, FHA-BP, FHA-OP, and FHA-DP specimens. After performing the hydrothermal treatment, it can be seen that a specific morphology with nano-scaled needle-like fine crystallites is deposited on the surface of hydrothermal FHA coatings, as shown in Figure 2b. The surface roughness of FHA coatings is about 7.3 ± 0.2 μm. It is recognized that surface with nano-crystalline features is important for promoting bioactivity, in vivo osseointegration, and biocompatibility of the metallic substrates [37] to use for hard tissue replacements and new bone regeneration [46,47]. Figure 2c–e shows the surface morphologies of FHA-BP, FHA-OP, and FHA-DP specimens, respectively. Compared with the original hydrothermal FHA coatings, we can see FHA-BP, FHA-OP, and FHA-DP specimens display similar microstructural feature and surface roughness (see Table 2). As a result, it represents that the SAM treatment by different organic alkyl-phosphonic acids did not affect surface morphologies of hydrothermal synthesized FHA surface coatings. In general, the monolayer is a two-dimensional molecular array, which is spontaneously organized by adsorption of amphiphilic organic molecules on a solid inorganic surface. Therefore, the SAM surface layer is hardly analyzed and observed by a SEM, and the surface of FHA-BP, FHA-OP, and FHA-DP specimens displays a similar microstructural feature to that of hydrothermal FHA coatings (Figure 2b) from the SEM observation. However, surface chemical states of surface elements for FHA-BP, FHA-OP, and FHA-DP specimens will be varied with performing the SAM-modified treatment. In following sections, the presence of surface monolayers on FHA layers will be further clarified and demonstrated by the static contact angle measurements and XPS analysis.

**Figure 3:**
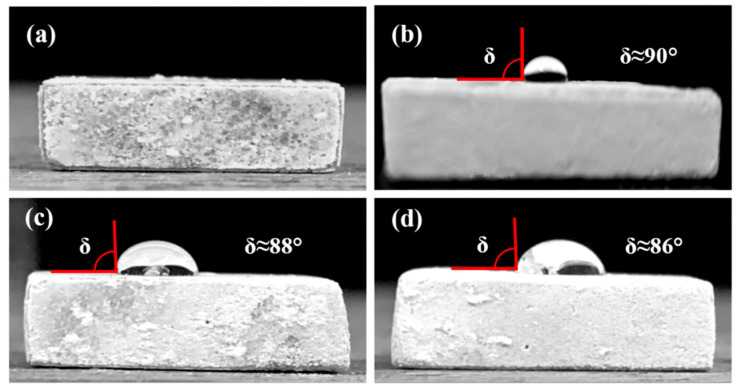
Hydrophilicity of the (**a**) hydrothermal FHA-coated AZ91D, SAM-treated (**b**) FHA-BP, (**c**) FHA-OP, and (**d**) FHA-DP specimens.

Figure 3 displays images of distilled water droplets on the surface of hydrothermal FHA coatings, FHA-BP, FHA-OP, and FHA-DP specimens by the assessment of static contact angles. Table 2 also lists the static contact angles (δ) of various specimens. We can see the contact angle of distilled water droplets on FHA coating surfaces is significantly less than 2°, as shown in Figure 3a. Generally, polar hydroxyl groups (OH^−^) and fluorine ions (F^−^) can promote a strong attractive interaction between FHA (Ca_10_(PO_4_)_6_(OH)F) and water molecules through the hydrogen bonds. Therefore, low static contact angle represents that the hydrothermal FHA coating displays a superior hydrophilicity result from its polar surface chemistry [48]. The hydrophilic surface is beneficial to cell adhesion, and the improvement of surface hydrophilicity also induces early cells attachment after implantation. Figure 3b–d illustrate the images of distilled water droplets on surface of FHA-BP, FHA-OP, and FHA-DP specimens, respectively. Table 2 lists the corresponding static contact angles of these specimens. Referring to Figure 2, surface morphologies of SAM surface-treated specimens almost display the same microstructural feature as the FHA coatings. However, the static contact angle of distilled water droplets on FHA-BP, FHA-OP, and FHA-DP specimens is significantly increased to about 86–90°, as illustrated in Figure 3b to Figure 3d. In well-ordered monolayers, the hydrophobic terminal methyl groups (–CH_3_) of the alkyl chains are oriented outward and effectively reducing access of the water drop to the surface [41]. Although the SAM-treated specimens display a high specific surface area with surface nano-scaled needle-like crystallites similar to FHA coatings (see Figure 2 for the SEM surface morphologies); however, the hydrogen bonds and capillary action on the SAM-treated surface will be further reduced because of the chemical bonding of a hydrophobic surface monolayer by BP, OP, and DP acids. Therefore, FHA-BP, FHA-OP, and FHA-DP specimens obviously show a surface chemistry with highly hydrophobicity in contrast to the hydrothermal FHA coatings. The lack of surface wettability for FHA-BP, FHA-OP, and FHA-DP specimens resulted from the organic alkyl-phosphonic (BP, OP, and DP) acids-derived monolayers directly bonded on the surface after performing the SAM surface treatment.

Considering the biological performances of a hydrophobic surface, Arima et al. [44] studied cellular interaction with a flat surface of SAM-treated gold-coated cover glasses and they indicated that both endothelial and epithelial cells can adhere and spread well onto the SAM-treated flat surface. However, the number of adherent cells decreased with increasing contact angles, and cell adhesion is reduced on the hydrophobic SAM. In addition to considering the effect of surface charge and chemical states on the biocompatibility of materials, surface topography is also an important affecting factor for cell adhesion [15]. It is reported that a biomimetic surface with micron-/nano-level topography has the potential to enhance cytocompatibility and biocompatibility [28,49]. Referring to the surface morphologies of SAM-treated specimens as illustrated in Figure 2c–e, these specimens still retain a surface feature with nanoneedles similar to the topography of bioactive FHA coatings. Therefore, it can be deduced that the hydrophobic alkyl-phosphonic acid-derived SAM specimens will keep corrosion resistance with desirable cellular interaction and biological performances.

### 3.2. XPS Analysis

**Figure 4:**
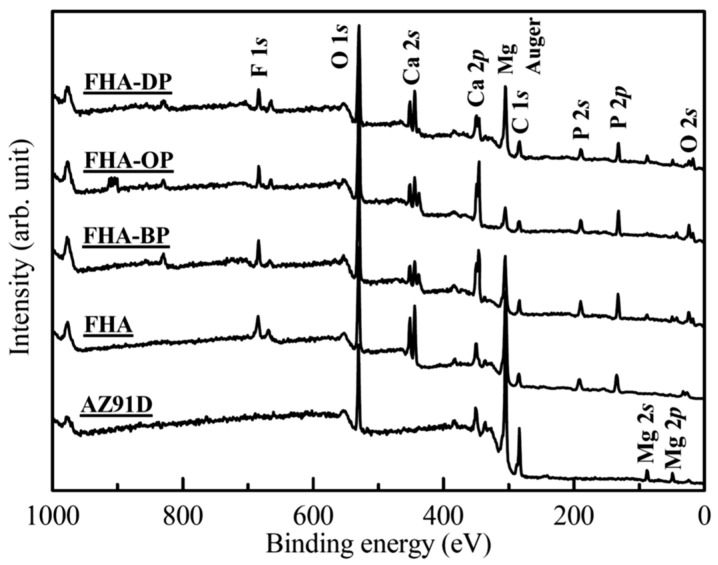
XPS survey spectra of the AZ91D Mg substrate, hydrothermal FHA surface coatings, FHA-BP, FHA-OP, and FHA-DP specimens.

The surface chemical status of Mg substrate with various surface treatments are determined by the XPS analysis. Figure 4 illustrates XPS survey spectra of these specimens, and the signals of C, O, and Mg elements are detected on the surface of AZ91D Mg alloy. Moreover, the signals of C, Ca, O, P, and F elements are detected on the FHA, FHA-BP, FHA-OP, and FHA-DP coating surfaces.

**Figure 5:**
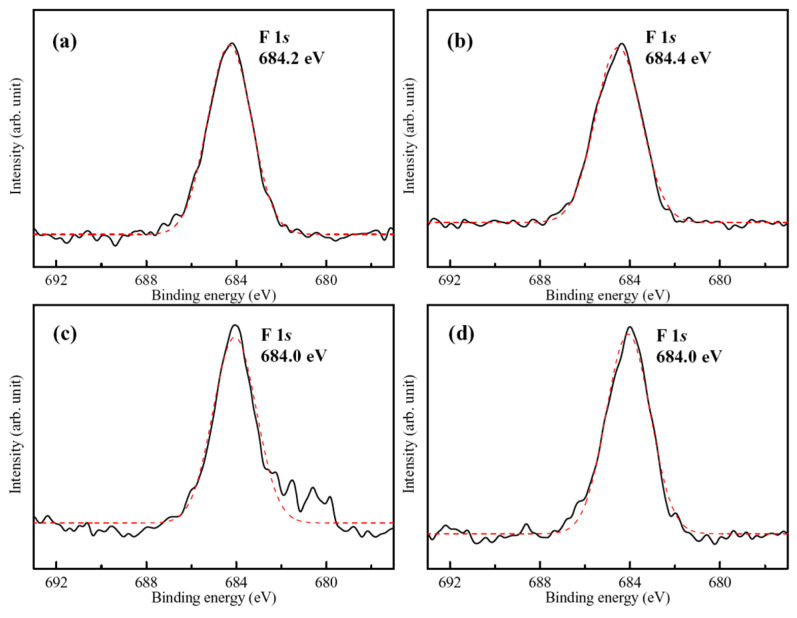
High-resolution XPS spectra of F 1*s* band for the (**a**) FHA coatings, (**b**) FHA-BP, (**c**) FHA-OP, and (**d**) FHA-DP specimens. (Solid lines are original XPS spectra of F 1*s* band, and red dash lines are Gaussian peak-fitting analysis results of F 1*s* band).

The representative high-resolution F 1*s* band and a curve-fitting result of the hydrothermally synthesized FHA coating is shown in Figure 5a. The binding energy (BE) of F 1*s* located at about 684.2 eV, which can be attributed to the substitution of F^−^-ions into FHA lattice (the BE of F 1*s* for F^−^-ions in FHA is about 684.2 ± 0.2 eV [32]). Figure 5b–d also display high-resolution F 1*s* spectra and curve-fitting results from the surface of SAM-treated FHA-BP, FHA-OP, and FHA-DP specimens, respectively. The BE values of F 1*s* band of the surface SAM-treated specimen are close to that of FHA coatings, and it also remains a single independent peak. The reason is that the surface grafted SAM is a nano-scaled monolayer, and X-ray photoelectrons can still directly penetrate the monolayer to detect and analyze the chemical state of F^−^ ions in FHA coatings. Therefore, it is recognized that performing self-assembly monolayers surface treatments will not change the original surface chemical states and lattice bonding structure of underlying FHA coatings. The molecular structures of the organic BP, OP, and DP alkyl-phosphonic acids with varying terminal methyl groups (–CH_3_) of the alkyl chains used in the experiment primarily consist of P=O and P-OH bonds. Therefore, high-resolution O 1*s* and P 2*p* bands are further analyzed to realize the surface chemical bonding and chemical shifts of SAM-treated specimens in the following paragraphs.

**Figure 6:**
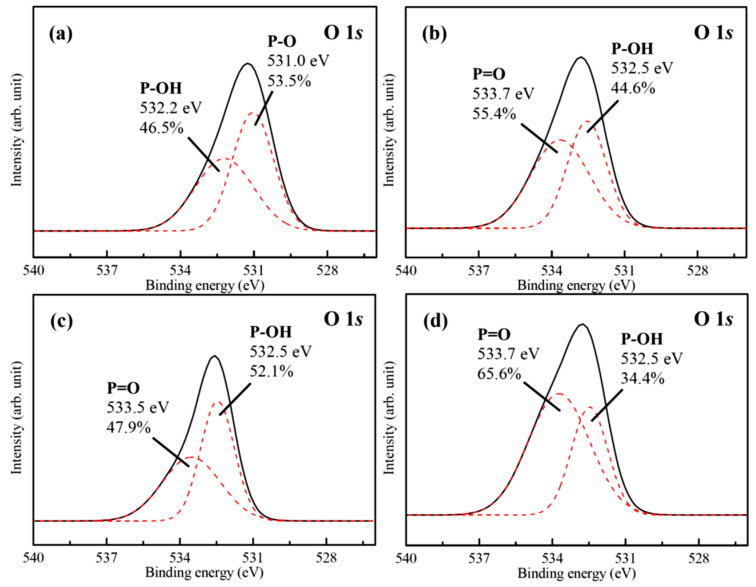
High-resolution XPS spectra of O 1*s* band for the (**a**) FHA coatings, (**b**) FHA-BP, (**c**) FHA-OP, and (**d**) FHA-DP specimens. (Solid lines are original XPS spectra of O 1*s* band, and red dash lines are Gaussian peak-fitting analysis results for various chemical bonds of O 1*s* band).

**Figure 7:**
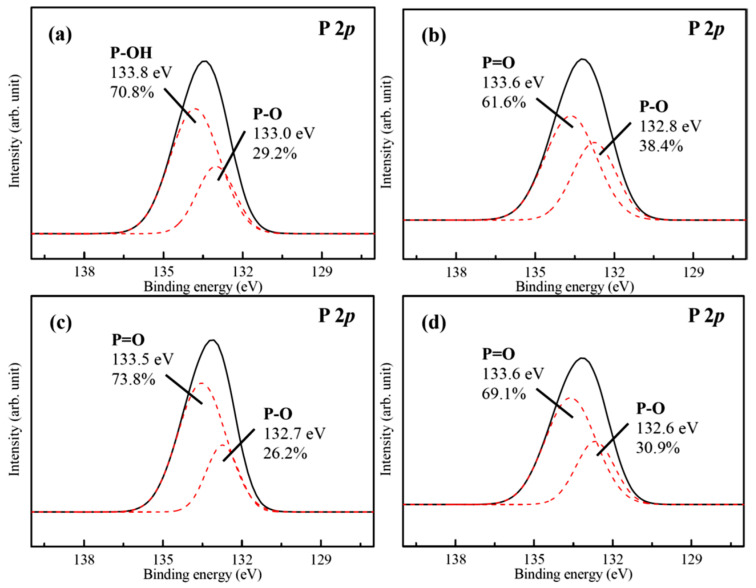
High-resolution XPS spectra of P 2*p* band for the (**a**) FHA coatings, (**b**) FHA-BP, (**c**) FHA-OP, and (**d**) FHA-DP specimens. (Solid lines are original XPS spectra of P 2*p* band, and red dash lines are Gaussian peak-fitting analysis results for various chemical bonds of P 2*p* band).

Figure 6 and Figure 7 show representative high-resolution O 1*s* and P 2*p* bands with curve-fitting results of the FHA coatings, FHA-BP, FHA-OP, and FHA-DP specimens. The corresponding O 1*s* band of the hydrothermal FHA coatings presented in Figure 6a consists of two components at BE = 531.0 eV and BE = 532.2 eV, which correspond to the presence of P-O bond (53.5%) in phosphate groups (PO_4_^3−^) and P-OH bond (46.5%) of the FHA lattice, respectively [50]. Although part of lattice bonding of OH^−^ groups is substituted by F^−^ ions, the presence of P-OH bond represents that the hydrothermal FHA coating sustains well-crystallized apatite crystal structure. Considering the SAM-treated FHA-BP, FHA-OP, and FHA-DP specimens, as shown in Figure 6b–d, the deconvoluted peaks located at BE = 532.5 eV and about BE = 533.5–533.7 eV express the presence of P-OH bond and P=O double bonds [41,42] of BP, OP, and DP alkyl-phosphonic acids monolayers, respectively. The head-group of phosphonic acids enables the molecule to anchor to the surface of FHA coating by the hydrogen bonding, and the surface of SAM-treated specimens will display hydrophobic terminal methyl groups (–CH_3_) to the surrounding solutions. In addition, the presence of P=O double bonds demonstrates that BP, OP, and DP alkyl-phosphonic acids monolayers are bonded on the surface of FHA. The P-OH bonds and P=O double bonds are both highly polar head groups of the alkyl-phosphonic acids (RPO(OH)_2_, R: alkyl group). It is reported that the alkyl-phosphonic acid-derived monolayers can be covalently bound to the hydroxide surface [41,42]. Therefore, the hydrophilic polar P-OH head groups can absorb on FHA (Ca_10_(PO_4_)_6_(OH)F) coatings with a strong chemical bonding by the SAM treatment. As a result, the terminal methyl groups (–CH_3_) of alkyl-chains are oriented outward and effectively provide a hydrophobic surface for FHA-BP, FHA-OP, and FHA-DP specimens.

Figure 7a shows the corresponding P 2*p* band of the hydrothermal FHA coatings. It constitutes of two components at BE = 133.0 eV and BE = 133.8 eV, which correspond to the presence of P-O bond (29.2%) in phosphate groups (PO_4_^3−^) and P-OH bond (70.8%) of the FHA lattice, respectively. Considering the SAM-treated FHA-BP, FHA-OP, and FHA-DP specimens, as shown in Figure 7b–d, we can see the P 2*p* band is also deconvoluted into two peak components located at about BE = 132.7 ± 0.1 eV and about BE = 133.5–133.6 eV corresponding to P-OH bond and P=O double bonds [50] after performing the SAM treatment with BP, OP, and DP alkyl-phosphonic acids monolayers. Referring to the XPS chemical composition analysis, O 1*s* and P 2*p* spectra (see Figure 6 and Figure 7), the significant P=O double bond and P-OH bond covalent bond reveal that the organic alkyl-phosphonic acid (BP, OP and DP) molecules can directly bond on the surface of FHA coatings.

### 3.3. Electrochemical Performances and Dissolution Behaviors in DMEM

**Figure 8:**
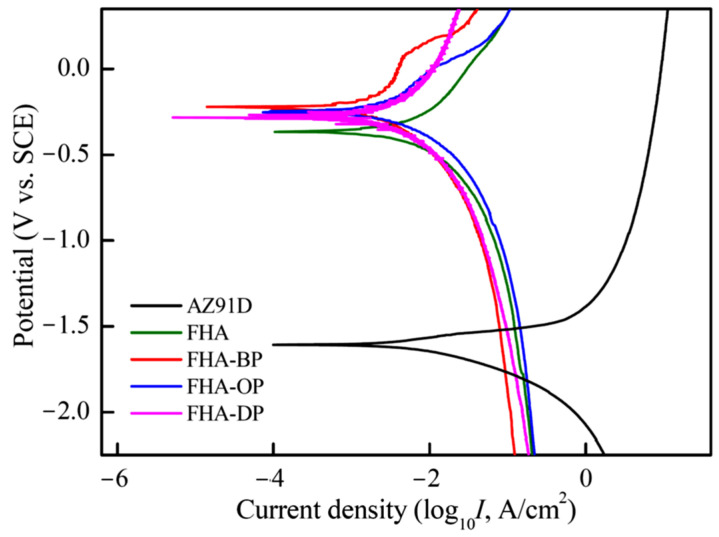
Potentiodynamic polarization curves of AZ91D alloy, hydrothermal FHA-coated AZ91D, SAM-treated FHA-BP, FHA-OP, and FHA-DP specimens tested in DMEM solution at 37 °C.

Figure 8 shows the electrochemical measuring results in terms of the potentiodynamic polarization curves of uncoated AZ91D substrate, hydrothermal FHA-coated AZ91D, FHA-BP, FHA-OP, and FHA-DP in the DMEM solution at 37 °C. The corrosion potential (*E*_corr._, V_SCE_) and the estimated corrosion current density (*I*_corr._, μA/cm^2^) are determined by Tafel extrapolation method. The corrosion current density is calculated from the measured polarization resistance (*R*_p_, kΩ-cm^2^), anodic Tafel slope (*β*_a_), and cathodic Tafel slope (*β*_c_) by the Stern–Geary equation. The electrochemical data for each test condition are the average value of at least three tests. The standard deviation in the *E*_corr._ values is within ± 25 mV. Table 3 lists these electrochemical analysis results obtained from potentiodynamic polarization tests in the DMEM solution.

As shown in Figure 8 and Table 3, uncoated AZ91D Mg alloy exhibits the most negative corrosion potential (*E*_corr._ = −1.61 V vs. SCE) of all the specimens. It is noted that the corrosion potential of FHA-coated AZ91D specimens is obviously increased to −0.37 V. Meanwhile, the corrosion current density is significantly decreased from 98.3 μA/cm^2^ of the uncoated AZ91D to 3.80 μA/cm^2^ of the FHA-coated specimen (see Table 3). The polarization resistance of FHA-coated specimens is also significantly improved to about 17.04 kΩ-cm^2^ compared to the uncoated AZ91D substrate (0.66 kΩ-cm^2^). We can see FHA-coated specimens show apparently higher corrosion potential, increased polarization resistance, and much lower corrosion current density. The formation of FHA coatings on the AZ91D substrate can supply an effective resistance to reduce the penetration and direct contact of the DMEM solution. It provides a substantial decrease in the instantaneous degradation rate of AZ91D Mg alloy in a corrosive environment.

Through performing the T-BAG SAM surface treatment for FHA-coated AZ91D, the corrosion potentials of all the FHA-BP, FHA-OP, and FHA-DP specimens are slightly improved to −0.23 V, −0.25 V, and −0.28 V, compared to the original FHA coating, respectively. Moreover, the reduction of corrosion current densities and the increased polarization resistances (see Table 3) of SAM-treated specimens represent that the corrosion reactions can be further suppressed with the direct chemical bonding of BP, OP, and DP alkyl-phosphonic acids monolayers on the FHA surface. It is noted that the FHA-DP specimen displays the lowest corrosion current density (0.51 μA/cm^2^) and the highest polarization resistance (*R*_p_ = 136.3 kΩ-cm^2^) of all the surface-treated specimens. As a result, it is recognized that long alkyl-chains of the dodecylphosphonic (DP) acid-treated SAM represents superior protective effects to enhance the anti-corrosion performance. Since the reduction of corrosion current densities and the improvement of polarization resistances are related to protective barriers on the surface against corrosive environments for the Mg substrate, the deposition of either hydrothermally synthesized FHA coatings, or the grafting of hydrophobic organic alkyl-phosphonic acids SAM can effectively promote a substantial decrease in the corrosion rate for AZ91D Mg alloy within the DMEM solution.

**Figure 9:**
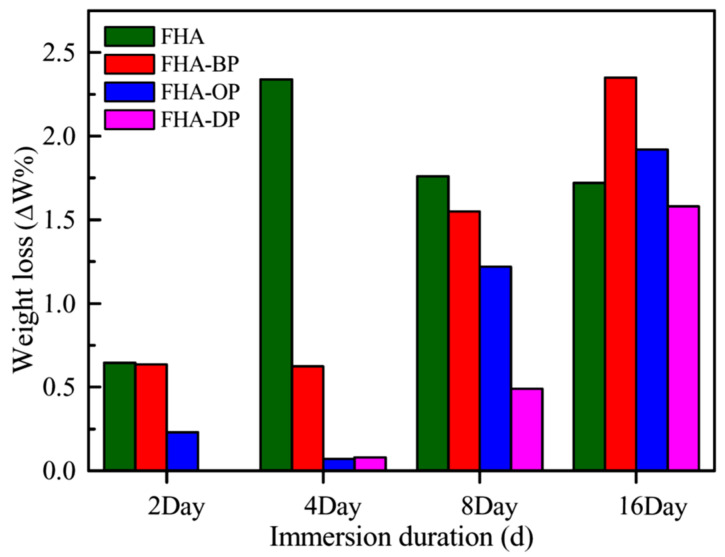
Weight loss of hydrothermal FHA-coated AZ91D, SAM-treated FHA-BP, FHA-OP, and FHA-DP specimens evaluated after immersion tests in the DMEM at 37 °C for 2, 4, 8, and 16 days.

Figure 9 shows a comparison of the weight loss of hydrothermal FHA-coated AZ91D, SAM-treated FHA-BP, FHA-OP, and FHA-DP specimens after a long-term immersion test in the DMEM solution for 2 to 16 days. The weight loss data for each experimental condition are the average of three specimens (*n* = 3). It represents that the weight loss increases with increasing immersion time for all specimens. For hydrothermal FHA-coated specimens, the weight loss obviously increases after 4 days of immersion. Since polar hydroxyl groups (OH^−^) and fluorine ions (F^−^) of the FHA crystal structure (Ca_10_(PO_4_)_6_(OH)F) can promote a strong attractive interaction between the FHA coating and water molecules by polar hydrogen bonds, it can be recognized that the surface of FHA-coated specimens displays superior hydrophilicity with low static contact angles resulted from its polar surface chemistry. The hydrophilic surface of FHA-coated specimens will gradually induce the penetration of the DMEM solution, and the increasing weight loss can arise from rapid corrosion reactions of the AZ91D substrates during the initial immersion period of time. The corroded Mg substrate causes the delamination of partly FHA coatings, and then results in a higher weight loss of specimens. With the extension of the immersion time for 8 to 16 days, the weight losses of the FHA-coated specimens are decreased and the values also tend to stabilize, which can be a result of their early large amount of delamination.

It is noted that the weight losses of SAM-treated FHA-BP, FHA-OP, and FHA-DP specimens are significantly decreased compared to that of FHA-coated AZ91D during the initial immersion period of time, especially for the 4 days of immersion. Even FHA-OP and FHA-DP specimens exhibit much lower weight losses of all conditions. The obvious reduction of weight loss for SAM-treated specimens in the initial few days can be attributed to the hydrophobic surface decreasing the penetration of solutions with less FHA coating delamination. Furthermore, the SAM-treated specimens still display lower weight loss than that of the FHA-coated specimens for a long term of immersion periods. The experimental results reveal that the grafting of self-assembled alkyl-SAM monolayers with a hydrophobic chemical state onto FHA coatings surface helps to inhibit the permeation corrosion effect of the DMEM solution to Mg substrates. The corrosion resistance of SAM-treated FHA-BP, FHA-OP, and FHA-DP specimens within the initial 2 to 4 days is significantly improved with their hydrophobic surface compared to the FHA-coated specimens. However, the corrosion weight loss increases with increasing immersion time for the SAM-treated specimens. Therefore, we deduce that the anti-corrosion effect of the alkyl-phosphonic acids monolayers will decrease with the reduction of hydrophobicity after a longer period of immersion time. In addition, the SAM treatment with longer alkyl-chains of the dodecylphosphonic acid to modify the surface of FHA-coated AZ91D (i.e., FHA-DP specimens) shows the most significant effects in reducing corrosion reactions and decreasing the weight loss.

Biodegradable Mg-based alloys have attracted extensive attention for their potential use as metallic implants for the applications of bone tissue regeneration engineering [5,6]. Surface modification of Mg implants with depositing protective coatings are applied for the reduction of their surface chemical activity for the purpose of improving corrosion resistance [26], and calcium phosphates, including stoichiometric HA and cations/anions-doped HA, are thought of appropriate ceramic coatings due to their bioactivities and excellent biocompatibility [20,27,28,29]. Relative studies have demonstrated that the deposition of FHA and Sr-doped HA (Sr-HA) coatings, which possess a micron-/nano-surface topography, can significantly improve the corrosion resistance of Mg substrates within the Kokubo’s simulated body fluid (SBF), and can also enhance cell viability on the FHA/Sr-HA-coated Mg alloys [28,32,33,51]. Furthermore, the chemical modified surface with the hydrophobic self-assembled monolayers is also a promising method to prevent the direct contact between corrosion ions and Mg alloys [41,42,52]. However, the anti-corrosion resistances and biological cell responses are dependent on the chemical stability of surface SAM [34,53]. The previous study investigated the combination effect of a ceramic coating with SAM on the anti-corrosion performances, and it was found that the organic SAM-treated Mg(OH)_2_ hybrid coatings effectively enhance both the chemical stability and anti-corrosion properties of Mg substrates [45]. Therefore, the deposition of organic alkyl-phosphonic acids-derived SAM directly bonded FHA hybrid coatings can not only improve the corrosion resistance, but also enhance the biocompatibility of AZ91D Mg alloys within the DMEM cell culture medium.

## 4. Conclusions

The following conclusions are drawn based on the above results and discussion:

(1) The FHA coating exhibits a super-hydrophilic surface chemical state with a static contact angle of less than 2° for the DMEM solution.
(2) The static contact angle of FHA coating surface is significantly increased after applying the SAM treatment with different alkyl chains of alkyl-phosphonic acids. SAM-treated FHA-coated AZ91D Mg alloy obviously display a stable surface chemistry with highly hydrophobicity resulted from the methyl terminal groups (–CH_3_) of the alkyl chains.
(3) XPS analysis results demonstrate that organic and hydrophobic 1-butylphosphonic (BP), 1-octylphosphonic (OP), and dodecylphosphate (DP) acids self-assembled monolayers (SAM) can be directly grafted to the hydrothermally synthesized FHA through the T-BAG method without affecting FHA surface morphologies.
(4) Both of bioactive FHA coating and SAM-treated FHA-BP, FHA-OP, and FHA-DP specimens significantly enhances the electrochemical corrosion resistance of AZ91D alloy in the DMEM solution.
(5) As a result of the immersion tests in the DMEM solution, the self-assembled various alkyl chains of alkyl-phosphonic acids hydrophobic monolayer on the FHA coating surface can inhibit the penetrative corrosion effect and decrease the weight loss after immersion. Longer alkyl-chains of the dodecylphosphonic acid (DP) monolayer display superior effect on improving corrosion resistance in the DMEM solution.
(6) Hydrophobic SAM-treated FHA surface can provide better corrosion resistance than the hydrophilic raw FHA-coated AZ91D specimens, which will be beneficial for cell attachment and proliferation in the further cell culture experiments.

## Figures and Tables

**Table 1:** Ion concentrations (mmol/L) and pH values for the used DMEM solution and human blood plasma.

	Na^+^	Cl^−^	K^+^	Ca^2+^	Mg^2+^	HCO_3_^−^	HPO_4_^2−^	SO_4_^2−^	pH
DMEM	155.3	115.7	5.3	1.8	0.8	44.1	0.9	0.8	7.4
Blood plasma	142	103	5	2.5	1.5	27	1	0.5	7.35–7.45

**Table 2:** The average surface roughness and the static contact angle of hydrothermal FHA coatings, FHA-BP, FHA-OP, and FHA-DP specimens.

	FHA	FHA-BP	FHA-OP	FHA-DP
Ra (μm) †	7.3 ± 0.2	7.1 ± 0.7	6.7 ± 0.6	6.9 ± 0.3
Static contact angle (δ)	<2°	90°	88°	86°

† Values were given as mean ± SD, and each value was the average of five tests (*n* = 5).

**Table 3:** Electrochemical parameters of the AZ91D substrate, hydrothermal FHA-coated AZ91D, SAM-treated FHA-BP, FHA-OP, and FHA-DP specimens tested in DMEM solution at 37 °C.

	*E*_corr._ (V vs. SCE)	*I*_corr._ (μA/cm^2^)	*R*_p_ (kΩ cm^2^)	*P*_i_ (mm/year)
AZ91D	–1.61	98.3	0.66	2.25
FHA	–0.37	3.80	17.04	0.09
FHA-BP	–0.23	1.62	48.36	0.04
FHA-OP	–0.25	1.97	32.58	0.05
FHA-DP	–0.28	0.51	136.3	0.01

## Data Availability

The original contributions presented in this study are included in the article. Further inquiries can be directed to the corresponding author.
